# Hospitalized patients’ pain experience before and after the introduction of a nurse-based pain management programme: a separate sample pre and post study

**DOI:** 10.1186/s12912-019-0362-y

**Published:** 2019-09-04

**Authors:** Gugsa Nemera Germossa, Ragnhild Hellesø, Ingeborg Strømseng Sjetne

**Affiliations:** 1 School of Nursing and Midwifery, Jimma University Institute of Health Sciences, Jimma University, 378 Jimma, Ethiopia; 2 Department of Nursing Sciences Institute of Health and Society Faculty of Medicine, University of Oslo, Oslo, Norway; 3 Division of Health Services, Norwegian Institute of Public Health, Oslo, Norway

**Keywords:** Pain management, Education, Nursing care, Inpatients

## Abstract

**Background:**

Many patients suffer from unrelieved pain in hospital settings. Nurses have a pivotal role in pain management. Hence, a nurse-based pain management programme may influence how hospitalized patients experience pain. In this study we investigated hospitalized patients’ experience of pain before and after the introduction of a two-component nurse-based pain management programme.

**Methods:**

A quasi-experimental design with a separate sample pretest-posttest approach was conducted on a convenience sample of 845 patients (Survey 1: *N* = 282; Survey 2: *N* = 283; Survey 3: *N* = 280) admitted to the four inpatient units (medical, surgical, maternity, and gynecology) of a university medical center. Data were collected at baseline, before the intervention six weeks after pain management education, and finally immediately after four months of rounding using an interviewer-administered questionnaire adopted from a Brief Pain Inventory and the American Pain Society Patient Outcome Questionnaire.

**Results:**

All the samples had similar sociocultural backgrounds. The proportion of patients who reported average moderate and severe pain intensity in the last 24 h were 68.8% in Survey 1, 72.8% in Survey 2 and then dropped to 48.53% in Survey 3 whereas those who reported moderate and severe pain intensity at the time of interview were 53.9% in Survey 1, 57.1% in Survey 2 and then dropped to 37.1% in Survey 3. The mean pain interference with the physical and emotional function was generally reduced across the surveys after the introduction of the nurse-based pain management programme. These reductions were statistically significant with *p* < 0.05.

**Conclusions:**

Though the survey findings must be taken with caution, they demonstrate that the nurse-based pain management programme positively influenced patient-reported pain intensity and functional interference at the university medical center. This shows the potential clinical importance of the programme for hospitalized patients.

## Introduction

The occurrence of pain symptoms is one of the primary reasons to seek healthcare in the general population. Pain is often distressing for patients if not adequately managed [1–3] and may lead to anxiety, depression, fatigue, a desire for death, escalated pain, poor quality of life, limitations in Activities of Daily Living (ADLs), poor compliance with treatment, and prolonged hospital stays [3–6].

Despite the availability of effective therapies, many patients continue to suffer from unrelieved pain in hospital settings [7]. As a result, international pain organizations have called for a strategy to improve pain management practices [2] that include pain assessment, appropriate use of analgesics, and proactive responses [8].

Even though pain management is the responsibility of every healthcare provider, it is the primary role of nurses. Nursing is an important caregiving situation, and pain management is an integral part of the practice of nursing. Left untreated, pain is considered professional misconduct or a violation of fundamental human rights [1–3]. Despite this, inadequately managed pain is highly prevalent, particularly in Ethiopian hospitals, due to a lack of appropriate care [9]. For example, it was reported that 80.1% of surgical patients at Jimma University Hospital were inadequately managed for pain [10]. Studies have revealed that a number of factors contribute to inadequate pain management: provider negligence, fragmented care, nurses’ lack of adequate knowledge of and attitudes towards pain, and the lack of a system that engages, empowers and motivates nurses [11–13].

Prior studies related to pain management were mainly focused on the prevalence of pain [14–16], the effectiveness of the educational programme [17–20], analgesic use [21], nonpharmacological therapies, and the description of interventions related to cancer and HIV pain [22, 23]. However, a study on the effectiveness of a nurse-led pain management intervention for patients with chronic pain that employed cognitive behavioral treatment showed an improvement in the reduction of pain intensity [24]. Yet, to our knowledge, there are no studies that investigate hospital patients’ pain intensity and interference across various units before and after the introduction of a nurse-based pain management programme. Thus, building on the existing system, we introduced a nurse-based pain management programme in an Ethiopian university hospital. The programme consists of intensive in-service nurse education aimed to enhance nurses’ knowledge of and attitudes towards pain [25], and a rounding routine to ensure the systematic monitoring of patients’ pain. Ultimately, the goal was to improve pain treatment practice and to measure the effectiveness of the programme on patients’ pain experiences on three occasions. The aim of this study was, therefore, to investigate the level of patient-reported pain experiences before and after the introduction of a two-component nurse-based pain management programme.

## Methods

### Study design and setting

A quasi-experimental design with a separate sample pretest-posttest approach was conducted at Jimma University Medical Center (JUMC) from 1 September 2016, to 15 July 2017.

### Participants

A convenience sample of 845 patients (Survey 1: *N* = 282; Survey 2: *N* = 283; Survey 3: *N* = 280) was invited to participate in this study. All patients who were admitted to the four inpatient units at the hospital (medical, surgical, maternity, and gynecology wards) were included if they filled the inclusion criteria. Patients had to have been hospitalized for at least 24 h, age ≥ 18 years, and have no known hearing impairment. Initial contact with patients was made through the ward’s head nurse or shifts leader. Participation in the study was voluntary. None of the patients approached declined participation.

### A nurse-based pain management programme

A nurse-based pain management programme is an intervention comprised of two components: education (to enhance nurses’ knowledge of and attitude towards pain) and organizational elements (to ensure the systematic monitoring of patients’ pain), with the goal of improving pain treatment. In the educational component, we provided two days of intensive in-service pain management training (16 h of face-to-face training), take-home reading assignments (self-learning), and refresher training four weeks later (8 h) for all nurses in the units. The education programme was arranged in groups and completed between 1 October and 15 November 2016. Each group was comprised of 30–40 nurses. The education sessions were delivered as per the pain management protocol developed by the research team, which was based on Ethiopia’s Federal Ministry of Health (FMOH) pain management guidelines [11] the World Health Organization’s (WHO) guidelines for pain management [26, 27]. Following the education programme, we introduced the second component, a rounding programme to educate staff nurses and nurse leaders in patient goal-oriented pain management [28]. The rounding programme consisted of an engagement orientation on how to organize and conduct rounding. The rounding programme lasted one day (8 h) for all staff nurses and a half day (four hours) for nurse leaders and supervisors. The content of the orientation included regular pain assessment using the numerical rating scale (NRS), charting in rounding logs when it was necessary to consult the physician, scripted dialogue with the patients, and how to assess patients using the four Ps: presence, pain, position, and personal needs. Rounding was structured in such a way that nursing directors, head nurses, and staff nurses proactively made regular and consistent visits to patients and performed scheduled tasks focused on pain management. When nurses visited patients during rounds, they introduced themselves and read the following script in either the Afaan Oromo or Amharic language (according to the patient’s language preference): “*We are going to do everything we can to help keep your pain under control. Your pain management is our number-one priority. Given your (condition, history, diagnosis, status), we may not be able to keep your pain level at zero. However, we will work very hard with you to keep you as comfortable as possible”* [4]*.* Staff nurses made subsequent visits every 2 h during the day (8,00 am–8,00 pm) and every 4 h at night (10,00 pm–6,00 am). During each visit, nurses assured patients of their availability and informed patients when they would return, asked patients to rate their pain levels, and recorded it in the pain log. If necessary, they repositioned the patient and checked for personal needs (toileting, getting out of bed, water).

More specifically, staff nurses systematically assessed every patient admitted to the four units up to ten times (every 2 h during the day and every 4 h during the night) in a 24-h period. Unless the patient was unconscious, sleeping or the bed was empty, the pain level was self-rated using the NRS and recorded in the pain log by the nurse. After each pain assessment, the nurse decided if the patient required a change in pain treatment regimen in collaboration with the treating physician, using the WHO pain ladder framework. Based on the collaborative decision, the nurse administered adjusted pain medication by the clock. “By the clock” means that the patient would be given analgesics regularly at a fixed interval of time-based on the known pharmacokinetics of the drug in use and that the next dose of analgesics would be adjusted before the effect of the previous dose had fully worn off. The rounding log was kept easily accessible for the healthcare provider’s review. In addition, nurses participated in the multidisciplinary team rounds and shared patient pain information.

Leadership rounding was performed daily by head nurses or clinical leaders (team leaders) and weekly by nursing directors. The leaders’ role was to motivate, facilitate, and provide positive feedback to the nurses. In addition, the head nurse-led weekly staff nurse discussions, and the nursing director led monthly discussions for head nurses and supervisors. Compliance with the rounding protocol was monitored twice weekly by nurse supervisors, using a review of the rounding log and discussion minutes.

### Measurements

Pain intensity and interference are regarded as reliable parameters to measure patients’ experiences of pain [29]. To measure the patient pain experience, we used a tool consisting of 18 items adapted from the Brief Pain Inventory (BPI) [30] and the American Pain Society Pain Outcome Questionnaire-Revised (APS-POQ-R) [31]. Items that were used to measure pain prevalence in the last 24 h (one item), pain treatment information (two items), and pain intensity (four items) were adopted from the BPI. Pain intensity/severity was measured on the 11-points NRS (from 0 = no pain to 10 = worst possible pain) with four scores: for pain present at the time of interview (“right now”), pain at its worst during the last 24 h, pain at its least over the past 24 h, and pain on average over the past 24 h. A pain severity/intensity level less than 4 is regarded as mild pain, greater than or equal to 4 and less than or equal to 6 is moderate, and greater than or equal to 7 is severe pain on the 0 to 10 NRS [1]. Eleven items used to measure pain interference (six items for physical functions and four items for emotional functions) were adopted from the APS-POQ-R. Patients were asked to rate their pain’s interference with physical functions such as activity in bed (sitting up, turning in bed), activity out of bed (walking, standing, squatting, use of wheelchair, dressing, etc.), deep breathing and coughing exercise (postoperative patients), sleeping (falling asleep, staying asleep), and relationships with others as well as interference with emotional function, in this case mood disturbance (anxious, depressed, frightened, feeling helpless). All functions were measured on an 11-point rating scale (0 = no interference, 10 = complete interference). Scores less than 3 indicate mild, greater than or equal to 3 and less than or equal to 4 indicate moderate, and greater than 4 indicate severe interference [1].

The tool was initially translated by healthcare professionals to Afaan Oromo and Amharic; linguistics and non-health care professionals then retranslated back to English. Then, all the translators came together to discuss the translated items. To check how each item was understood, a cognitive interview was conducted with five people of varying backgrounds [32]. Finally, the tool was tested on 35 patients from various units to clarify words and the sequence of the items. We have also collected information on admission unit (the type of unit), sociodemographic characteristics (age, sex, address), and socioeconomic variables (educational level, occupation, monthly income). The principal investigator collected the completed questionnaires from the data collectors daily and stored them in locked cabinets.

### Data collection procedure

Data was first collected at baseline (Survey 1), again six weeks after the educational programme (Survey 2), followed by a third survey immediately after four months of rounding (Survey 3). The data were collected by trained nurses through a structured face-to-face interview that lasted approximately 40–45 min.

### Data analysis

All surveys were assessed for missing or incomplete data before being analyzed (survey 1: 26, survey 2: 24, survey 3: 13). Data were analyzed using Statistical Package for the Social Sciences (SPSS) version 20.1 (IBM SPSS Statistics for Windows, Armonk, NY). Descriptive statistics (i.e., mean, standard deviations, range, frequency) were calculated for patient characteristics (age, income), pain severity, and interference response items. Reduction in the sample means score was calculated by subtracting the mean value of survey 3 from the mean value of survey 1, dividing it by the survey 1 value, and multiplying by 100. Differences between the mean pain intensity and interference scores at baseline (Survey 1), six weeks after the in-service educational program (Survey 2), and immediately after four months of rounding (Survey 3) were analyzed using a one-way Analysis of Variance (ANOVA) with a post-hoc Bonferroni test. The significant differences between the surveys were declared at *p* < 0.05.

## Results

### Sample characteristics

**Fig. 1:**
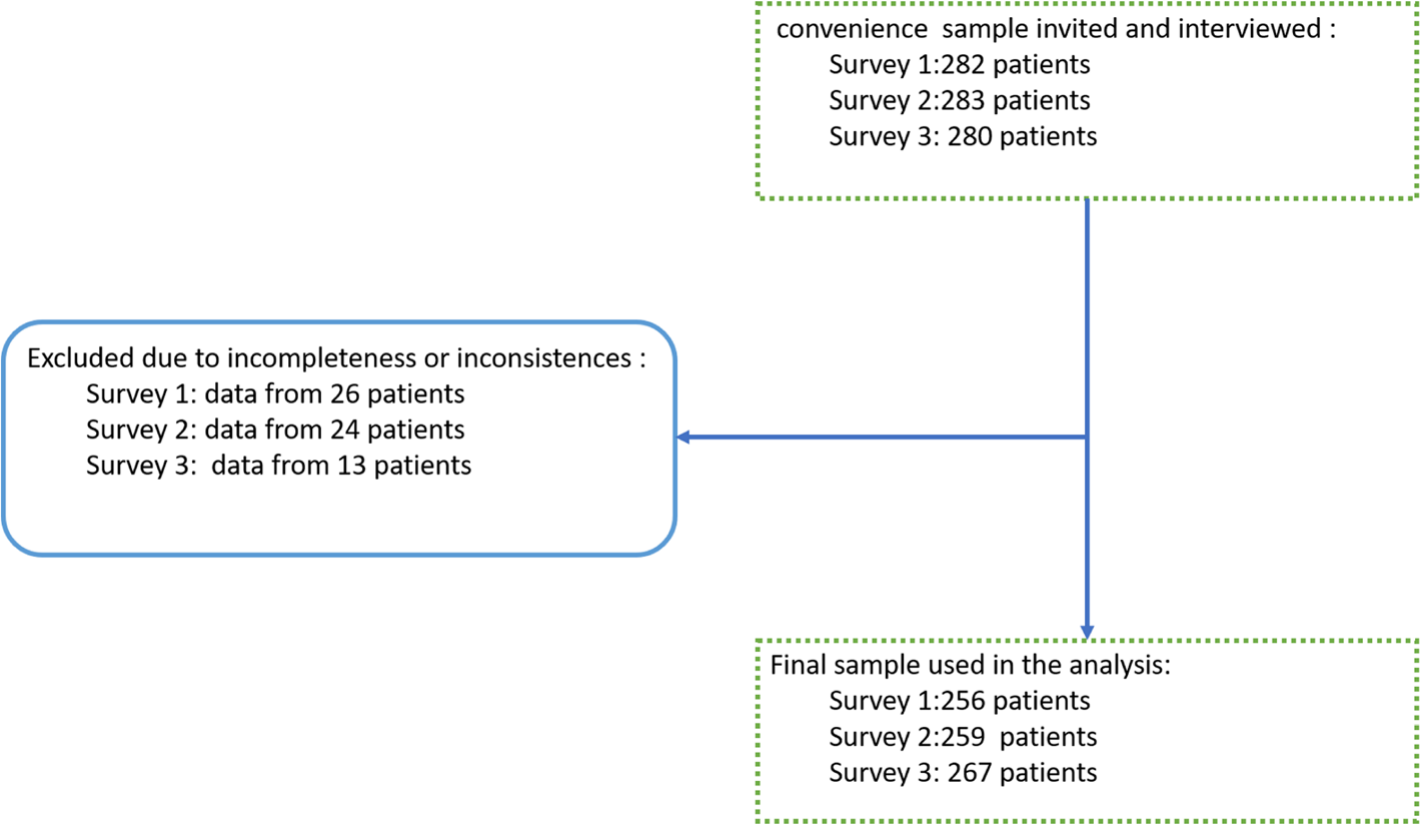
Schematic representation of subject recruitment. Data was collected three times from three different samples. First at baseline (Survey 1), again six weeks after the educational programme (Survey 2), and for the third time immediately after the four months of rounding (Survey 3)

Of the 845 eligible patients, 782 patients’ complete responses from (Survey 1: *N* = 256; Survey 2: *N* = 259; Survey 3: *N* = 267) were analyzed (Fig. 1). There were no differences in the mean ages of the respondents (Survey 1: mean age = 38.1 (SD ± 16.2); Survey 2: mean age = 37.4 (SD ± 15.2); Survey 3: mean age = 37.9 (SD ± 5.2)). Though the percentage of missing values for income was high (30.4% for Survey 1, 42.8% for Survey 2, and 60% for Survey 3), the median monthly income in Ethiopian currency was 1000 birr for all surveys: the interquartile range was 1054 birr for Survey 1, 1500 birr for Survey 2, and 1500 birr for Survey 3. When converted to US currency, 50% of respondents earned just over one US dollar per day (1 US dollar ≈ 27.91 Ethiopian birr). There were no statistically significant differences in sociocultural characteristics between the three samples, and there were no reports of new disease epidemics in the area. As shown in Table 1, except for the unit of admission, there was no difference in the distribution of the sample characteristics by survey period.

**Table 1:** Sample characteristics

Sample characteristics	Survey 1 Number (%)	Survey 2 Number (%)	Survey 3 Number (%)	*p*-value ^a^		
				Survey 1 vs Survey 2	Survey 1 vs Survey 3	Survey 2 vs Survey 3
Gender	(*N* = 256)	(*N* = 259)	(*N* = 267)			
Male	125 (48.8)	139 (53.7)	134 (50.2)	0.274	0.757	0.426
Female	131 (51.2)	120 (46.3)	133 (49.1)			
Address	(*N* = 247)	(*N* = 237)	(*N* = 263)			
Urban	169 (68.4)	164 (69.2)	173 (65.8)	0.808	0.528	0.418
Rural	78 (31.6)	73 (30.8)	90 (34.2)			
Educational level	(*N* = 253)	(*N* = 253)	(*N* = 266)			
Had no formal education	151 (59.7)	150 (59.3)	171 (64.3)	0.928	0.282	0.243
Had formal education	102 (40.3)	103 (40.7)	95 (35.7)			
Occupation	(*N* = 253)	(*N* = 253)	(*N* = 266)			
Farmer	151 (59.9)	133 (51.8)	148 (55.4)	0.168	0.366	0.074
Government employee	28 (11.1)	44 (17.1)	29 (10.9)			
Self-employed	36 (14.3)	41 (16.0)	35 (13.1)			
Unemployed	37 (14.7)	39 (15.2)	55 (20.6)			
Unit of admission	(*N* = 256)	(*N* = 259)	(*N* = 267)			
Surgical	133 (52.0)	104 (40.2)	98 (36.7)	0.008	0.001	0.831
Medical	86 (33.6)	89 (34.4)	101 (37.8)			
Gynaecology	20 (7.8)	34 (13.1)	34 (12.7)			
Maternity	17 (6.6)	32 (12.4)	34 (12.7)			

^a^ Critical value when proportions were compared using WINPEPI, using a comparison of two independent samples

### Pain treatment

Most patients received anti-pain medication intramuscularly and/or intravenously. In Survey 2, the proportion of patients treated with a pharmacological agent increased by 14.1% compared with Survey 1, by 4.8% in Survey 3 compared with Survey 2, and by 19.7% in Survey 3 compared with Survey 1. Prayers, massage, and cold or hot application were commonly reported nonpharmacological pain therapies provided by patient attendants (Table 2).

**Table 2:** Patient-reported pain treatment method and route of pain medication administration

Pain treatment	Survey 1 (*N* = 256) Number (%)	Survey 2 (*N* = 259) Number (%)	Survey 3 (*N* = 267) Number (%)
Method of treatment			
Pharmacological	161 (62.9)	186 (71.8)	201 (75.3)
Non- pharmacological	13 (5.1)	8 (3.1)	4 (1.5)
Mixed	72 (28.1)	57 (22.0)	56 (21.0)
None	8 (3.1)	8 (3.1)	6 (2.2)
Missed data	2 (0.8)	0	0
Route of pain medication			
Parenteral (IM/IV)	105 (45.1)	124 (51.0)	137 (53.7)
Oral	44 (18.9)	54 (22.2)	44 (17.3)
Both	84 (36.1)	65 (26.7)	74 (29.0)

### Pain intensity

Table 3 shows the samples’ mean pain intensity in the first, second, and third surveys. The results of all three surveys show that patients generally had moderate to severe pain. However, the mean pain intensity levels were generally reduced across the survey period. This reduction was statistically significant between the second and first survey as well as between the third and first survey for the worst pain, least pain, and pain “right now”. Reduction in the samples’ mean scores for pain intensity at its average during the last 24 h was statistically significant between all three surveys. In the third survey, the sample means’ pain intensity was reduced by 27.6% at for pain at its worst, 23.8% for pain at its least, 25.5% for pain at its average over the last 24 h, and current pain by 29.3% compared with Survey 1. Even though the proportion of patients who reported pain in the last 24 h generally decreased after the intervention, the proportion of patients who experienced pain in the last 24 h in the second survey was slightly higher (94.5%) than in the first survey (93%). However, immediately after four months of rounding (in the third survey), the proportion was reduced to 87.3%.

**Table 3:** The sample mean pain intensity scores

Pain intensity	Survey 1 Mean (SD)	Survey 2 Mean (SD)	Survey 3 Mean (SD)	*p*-value ^a^		
				Survey 1 vs. Survey 2	Survey 1 vs. Survey 3	Survey 2 vs. Survey 3
Your pain at its worst in the last 24 h	5.8 (2.6)	5.3 (2.0)	4.2 (1.8)	0.069	0.000	0.001
Your pain at its least in the last 24 h	4.2 (2.4)	4.0 (2.0)	3.2 (1.6)	0.627	0.000	0.001
Your pain on average in the last 24 h	4.7 (2.2)	4.2 (1.2)	3.5 (1.4)	0.008	0.000	0.001
How much pain you have right now	4.1 (2.8)	3.8 (2.0)	2.9 (1.9)	0.387	0.000	0.001

^a^ *P*-value using one-way ANOVA with post hoc test

The results of all three surveys show that patients generally had moderate to severe pain when asked for “average pain in the last 24 hours” and “pain right now”. However, as indicated in Table 4, the proportion of patients with severe pain was generally reduced across the survey period.

**Table 4:** The proportion of patients with mild, moderate and severe pain when asked for average pain in the last 24 h and pain “right now” by survey period

Severity of pain		Survey 1	Survey2	Survey 3
Average pain	N	237	247	233
	Mild	31.2	27.1	51.5
	Moderate	47.3	69.2	46.4
	Severe	21.5	3.6	2.1
Pain at the time of the interview (“right now”)	N	256	259	267
	Mild	46.1	42.9	62.9
	Moderate	32.4	49.4	34.5
	Severe	21.5	7.7	2.6

### Pain interference

In all three surveys, the score for the mean pain interference scales indicates moderate to severe interference with both physical and emotional functions. As shown in Table 4, only minor differences were observed in the level of patient-reported pain interference between Survey 1 and Survey 2. Apart from activities out of bed, a statistically significant reduction in pain interference with physical functions was observed between both the first and third surveys and the second and third surveys. On the other hand, the mean level interference with relationship and negative feelings (anxious, depressed, frightened, and helpless) significantly decreased in Survey 2 and Survey 3 compared with Survey 1. However, the reduction in feeling helpless occurred between the third and second surveys (Table 5).

**Table 5:** The samples’ mean scores on pain interference with physical and emotional function

Pain interference with	Survey 1 Mean (SD)	Survey 2 Mean (SD)	Survey 3 Mean (SD)	*p*-value		
				Survey 1 vs. Survey 2	Survey 1 vs. Survey 3	Survey 2 vs. Survey 3
Physical functions						
Activity in bed	4.6 (3.4)	4.6 (3.1)	3.9 (2.8)	1.000	0.026	0.021
Activities out of bed	5.0 (3.6)	4.9 (3.1)	4.4 (3.1)	1.000	0.073	0.170
Falling asleep	4.3 (3.1)	4.3 (2.8)	3.5 (3.1)	1.000	0.014	0.015
Staying asleep	4.3 (3.1)	4.1 (3.0)	3.7 (3.2)	1.000	0.044	0.379
Deep breathing and coughing	3.4 (3.2)	3.1 (3.4)	1.2 (2.2)	1.000	0.000	0.001
Emotional functions						
Relationships with others	3.6 (3.2)	3.0 (3.1)	2.8 (2.9)	0.089	0.010	1.000
Anxious	5.4 (3.1)	5.3 (2.7)	4.3 (3.0)	1.000	0.000	0.001
Emotion (Depression)	5.2 (3.0)	5.2 (2.7)	4.3 (3.1)	1.000	0.001	0.003
Frightened	4.9 (3.2)	5.0 (3.0)	4.1 (3.4)	1.000	0.019	0.011
Helplessness	3.7 (3.4)	3.8 (3.3)	3.1 (3.9)	1.000	0.142	0.036

^a^ *P*-value using one-way ANOVA with post hoc test

## Discussion

In this study, we investigated patients’ pain experiences before and after the introduction of a nurse-based pain management intervention. The goal of the intervention was to improve pain treatment practices and provide timely and optimal treatment. The overall findings show that patients reported less pain intensity as well as functional interference after the intervention. The mean pain intensity level at its worst, least, and on average in the last 24 h and at the time of survey (“right now”) was generally reduced in the third survey immediately after four months of rounding. On the other hand, the proportion of patients who reported pain in the last 24 h was reduced from 93% in Survey 1 to 87.3% in Survey 3. Similarly, the mean pain interference with physical and affective functions was also greatly reduced. These reductions can be attributed to better pain management following the intervention. This could have prevented patients from severe discomfort and impaired physiological homeostasis such as depressed mood, fatigue, limitation of ADLs, and anxiety [3–6, 33]. The intervention is in line with the WHO [34], the APS [8], the Joint Commission on Accreditation of Healthcare Organizations (JCAHO) [35], the (FMOH) [11] guidelines. These guidelines recommend regular pain assessment and appropriate use of pain therapies.

Pain intensity and interference with function are important parameters in the evaluation of the effectiveness of pain management interventions on the part of patients [29, 36] and patient responses to pain-producing medical procedures [37]. The findings of the current study show the degree to which patients received the essential elements of pain management: pain assessment, aligning analgesics with the patient’s pain level, and consistent monitoring. Compared with the results in Survey 1, pain intensity level (at present, at least, at worst, and on average), and the level of pain interference with physical and emotional function significantly decreased in the second and third surveys. Though there are no similar studies with which to compare, the findings are in accordance with results from other earlier, related studies, including nurse-based pain management programmes [24], pain educational programmes [17–20], and postoperative pain management programmes [22, 23] on chronic pain and its interference with physical and emotional function.

Even though the current study showed a significant positive impact on the mean pain intensity, mean functional interference level, and the proportion of patients who reported pain in the last 24 h (93% in Survey 1 vs. 87.3% in Survey 3), the proportion of patients who reported pain in the last 24 h at the end of intervention was still high when compared with the results of prior prevalence studies in a German teaching hospital (63%) [14], and in Chicago, USA (59%) [38]. Even though the proportion of patients regularly assessed for pain and treated with anti-pain medication through different routes significantly increased, the findings of the current study imply that a larger number of patients still suffer from manageable pain. This could be due to limitations on the availability of anti-pain medication. There is also a possibility that increased attention to pain management from nurses may have given the patients higher expectations towards pain relief and thereby impacted patient responses to Surveys 2 and 3.

The commonly-used pain medications in the study hospital were tramadol, Non-Steroidal Anti-Inflammatory Drugs (NSAID) (paracetamol, ibuprofen, diclofenac, indomethacin), and, rarely, pethidine. However, morphine, the gold standard indicator of adequate pain management, was either not regularly available or not appropriately used. The explanation of medication choice may be linked to various reasons. Those patients who came from districts where community-based health insurance was established and was legally allowed to receive healthcare services free of charge (the poor, pregnant mothers) automatically received all available pain medications in the hospital. However, patients outside this category paid out of pocket. In addition to pharmacological agents, some patients also used nonpharmacological interventions such as massage, prayer, or hot or cold application. On the other hand, the fact that the average monthly income of patients was just over one US dollar per day means that many patients who pay out of pocket may not be able to afford medication or get the medication in a timely fashion. Another possible reason could be the nature of pain. Acute pain is a protective warning signal indicating inflammatory or traumatic tissue damage, whereas chronic pain is a disease per se [2]. Thus, the patient may be in unnoticed acute pain due to missed visits or inadequately managed chronic pain at the time of data collection. However, these situations were the same in the three observation periods.

In a complex intervention that consists of an educational programme and rounding, it is difficult to attribute the contribution of each specific component to the final results [39]. The educational programme improved nurses’ knowledge of and attitudes towards pain [25] and was a cornerstone for an evidence-based pain management practice. This could inspire individual nurses to practice proper pain management. Thus, the findings from the second survey, which occurred after the educational programme, could be related to these changes in pain intensity except for average pain in the last 24 h. Rounding, on the other hand, further improved pain treatment by systemizing nurses’ care delivery practices in pain management routines. Hence, changes in pain intensity and interference in the final survey are most likely due to the combined effect of both the educational program and rounding. Within the scope of this study, we could only determine the impact of the entire intervention against the baseline result for patient-reported pain intensity and functional interference, though these findings must be taken with caution. The educational components of the nurse-based pain management programme upgraded nurses’ understanding of pain management, which seemed to help them carry out pain assessments, align analgesics to pain severity levels and monitor patient responses to treatment more confidently. Rounding in this program helped nurses apply their knowledge by organizing pain management practices so that patients were assessed regularly and treated for pain based on the WHO pain ladder.

### Strengths and limitations

The current findings indicate the potential benefits of a nurse-based pain management programme (in-service educational programme and rounding) for hospitalized patients. However, several factors may limit the findings of the current study. One limitation could be attributed to the study design, the use of a non-randomized design without a control group. Initially, we planned to employ a quasi-experimental design with a control group. This was however complicated by a period of public unrest followed by a declaration of a state of emergency, which made it impossible to travel between the intervention and control sites, and our plans to use a control group had to be abandoned. Another limitation is the possibility that nurses may have gained additional knowledge by interacting with medical professionals, thereby improving their practices, resulting in the possibility of physician-initiated pain treatment rather than nurse-initiated pain treatment through consultation. Given all these factors, a simple pre-post study design with three measurement points on separate samples is inadequate for causal inference. Further randomized, multicenter studies are necessary before attributing a nurse-based pain management programme to changes in patient-reported pain intensity and interference. Even though we have no data on the duration of hospitalization or types of procedures the patients had undergone during their hospital stays, it should be noted that this might have influenced the severity of pain.

## Conclusion

The current study provides empirical evidence that a nurse-based pain management programme (in-service education and rounding) significantly improved patient-reported pain intensity and interference. The instruments used in this survey could be used for monitoring pain management practices at regular intervals to ensure that the changes are sustainable. The findings also imply the need for educational programmes to improve nurses’ technical capacity in in-hospital nursing care. In addition, the phased intervention approach we have used in this study can easily be applied to nursing practices other than pain management, to improve patient-reported outcomes.

## Data Availability

Due to the NSD’s (Norwegian Centre for Research Data) policy, data from this research will not be shared to ensure data confidentiality but can be made available based on official request.

## Abbreviations

ANOVA: Analysis of Variances
APS: American Pain Society
APS-POQ-R: American Pain Society Pain Outcome Questionnaire-Revised
BPI: Brief Pain Inventory
FMoH: Ethiopia’s Federal Ministry of Health
IM: Intramuscular
IV: Intravenous
JCAHO: Joint Commission on Accreditation of Healthcare Organizations
JUMC: Jimma University Medical Center
NRS: Numerical Rating Scale
NSAID: Non-Steroidal Anti-Inflammatory Drug
SD: Standard Deviation
SPSS: Statistical Package for the Social Sciences
WHO: World Health Organization
